# Generalized Arterial Calcification of Infancy Type 1 (GACI1): Identification of a Novel Pathogenic Variant (c.1715T>C (p.Leu572Ser))

**DOI:** 10.3390/diagnostics11061034

**Published:** 2021-06-04

**Authors:** Gaetano Pietro Bulfamante, Laura Carpenito, Emma Bragantini, Silvia Graziani, Maria Bellizzi, Christoph Peter Bagowski, Moneef Shoukier, Francesca Rivieri, Massimo Soffiati, Mattia Barbareschi

**Affiliations:** 1 Human Pathology and Medical Genetic Unit, Department of Health Sciences, San Paolo Hospital, University of Milan, 20142 Milan, Italy; gaetano.bulfamante@unimi.it; 2 School of Pathology, University of Milan, 20122 Milan, Italy; 3 Department of Pathology, Santa Chiara Hospital, 38122 Trento, Italy; emma.bragantini@apss.tn.it (E.B.); mattia.barbareschi@apss.tn.it (M.B.); 4 NICU, Santa Chiara Hospital, 38122 Trento, Italy; silvia.graziani@apss.tn.it; 5 Department of Pediatrics, Santa Chiara Hospital, 38122 Trento, Italy; maria.bellizzi@apss.tn.it (M.B.); massimo.soffiati@apss.tn.it (M.S.); 6 Prenatal Medicine Münich, Department of Molecular Genetics, University Hospital, Aiblingerstr. 8, 80639 Münich, Germany; bagowski@praenatal-medizin.de (C.P.B.); shoukier@praenatal-medizin.de (M.S.); 7 Medical Genetic Service, Santa Chiara Hospital, 38122 Trento, Italy; francesca.rivieri@apss.tn.it

**Keywords:** GACI, mutations, *ENPP1* gene, pathogenic variant, autopsy, arterial calcifications

## Abstract

Generalized Arterial Calcification of Infancy (GACI) is a rare disease inherited in a recessive manner, with severe and diffuse early onset of calcifications along the internal elastic lamina in large and medium size arteries. The diagnosis results are from clinical manifestations, imaging, histopathologic exams, and genetic tests. GACI is predominantly caused by biallelic pathogenic variant in the *ENPP1* gene (GACI1, OMIM#208000) and, to a lesser extent, by pathogenic variants in the *ABCC6* gene (GACI2, OMIM#614473). We present a novel variation in the *ENPP1* gene identified in a patient clinically diagnosed with GACI and confirmed by genetic investigation and autopsy as GACI type 1. The sequence analysis of the patient’s *ENPP1* gene detected two heterozygous variants c.1412A>G (p.Tyr471Cys) and c.1715T>C (p.Leu572Ser). The variant c.1715T>C (p.Leu572Ser) has not been described yet in the literature and in mutation databases. A genetic analysis was also carried out for the parents of the newborn; the heterozygous pathogenic variant c.1412A>G (p.Tyr471Cys) was detected in the mother’s *ENPP1* gene, and a sequence analysis of the father’s *ENPP1* gene revealed the novel heterozygous variant c.1715T>C (p.Leu572Ser)**.** Our results showed that the variant c.1715T>C (p.Leu572Ser) may have a pathogenic role in the development of GACI type1 (GACI1, OMIM#208000), at least when associated with the pathogenic c.1412A>G (p.Tyr471Cys) variant. The identification of novel mutations potentially enabled genotype/phenotype associations that will ultimately have an impact on clinical management and prognosis for the disease.

## 1. Introduction

Generalized Arterial Calcification of Infancy (GACI) is an extremely rare recessive genetic disorder, with early onset of severe and diffuse calcifications along the internal elastic lamina in large- and medium-sized arteries. It is characterized by narrowing of the vessels with proliferation of the intima, leading to arterial stenosis [1,2]. As a consequence, affected individuals present with arterial hypertension, respiratory distress, cyanosis, and heart failure. GACI has a very poor prognosis; death by cardiovascular complications occurs mostly within the first year of life, due to the involvement of the coronary arteries [1,3]. The disease starts in utero, and it is possible to detect an increase in brightness of the main fetal arteries on a prenatal ultrasound already at 18 weeks of gestation [2].

Approximately 300 cases of GACI have been reported in the literature. The prevalence is unknown, while an estimated frequency of 1:566,000 has been suggested [4].

This disease involves multiple systems, especially the cardiovascular system, with possible involvement of the central nervous system [3]. Generally, GACI can present as fetal distress, polyhydramnios, hydrops fetalis, heart failure, hypertension, cardiomegaly, visceral effusion, respiratory distress, cyanosis, or a reduced peripheral pulse. A possible association between GACI (GACI, type 2) and some clinical manifestations of pseudoxanthoma elasticum (PXE) has been reported in the literature (such as angioid streaks of the retina and papular skin lesions) [1].

The diagnosis of GACI results from clinical manifestations, imaging, histopathologic exams, and genetic tests, confirmed eventually by autopsy [1,3]. Histopathologic findings include calcium hydroxyapatite deposition, which is visible in the internal elastic lamina, and diffuse fibrointimal hyperplasia with large- and medium-sized arteries showing a narrowed lumen [1].

It is important to obtain a diagnosis as early as possible in order to start treatment (vascular calcifications can be detected with a prenatal ultrasound).

Two types of GACI have been described. GACI type 1 (GACI1) is caused by biallelic inactivating mutations in the *ENPP1* gene (OMIM#173335), which are detected in about 70% of all patients. A minority of affected individuals (about 9%) have biallelic pathogenic variants in the *ABCC6* (ATP-binding cassette subfamily C number 6) gene, causing GACI type 2 (GACI2), while a few cases likely carry mutations in genes that have yet to be identified [1,5]. The ABCC6-transported substrate regulates the calcification processes [6].

*ENPP1* is located on chromosome 6q23.2 and encodes ectonucleotide pyrophosphatase phosphodiesterase 1 (NPP1), which produces extracellular inorganic pyrophosphate (PPi) and AMP from extracellular ATP [1]. PPi inhibits the deposition of hydroxyapatite crystals [2]. A decreased production of PPi consequent to mutations in *ENPP1* leads to an easier hydroxyapatite deposition. Besides GACI1, biallelic mutations in this gene cause also hypophosphatemic rickets, and mutations in *ABCC6* cause Pseudoxanthoma elasticum (PXE; OMIM#264800) [3].

It is still unknown whether a genotype–phenotype correlation exits [5]. Several cardiovascular phenotypes are possible, and clinical variability with different outcomes has been reported even in siblings with the same mutation [2].

In this article, we report on a novel variant in the *ENPP1* gene identified in a patient diagnosed with GACI type 1.

## 2. Clinical Data

The patient was the third child of nonconsanguineous parents without a family history of the disease. The pregnancy was uneventful, with normal ultrasound scans until the 28th gestational week, with a progressive development of nonspecific signs of intrauterine heart failure (severe polyhydramnios with hydrops fetalis, ascites, ductus venosus blood flow alterations, and fetal cardiac blood flow abnormalities). Therefore, at 28 weeks of gestation, an emergency caesarean section was performed without steroid prophylaxis. Maternal blood tests were within the normal range.

At birth, the newborn presented with hydrops fetalis requiring intubation (Apgar Index 2-4-7; Birth weight 1685 g, >97°). The newborn was admitted to the neonatal intensive care unit where he underwent an echocardiography that showed severe hypokinesia and left ventricular dilation and a thoracic ultrasound that revealed an important bilateral pleural effusion. A bilateral pleural drainage was inserted, and the citrine fluid was removed. Previous blood tests and clinical data were suggestive of suspected sepsis (CRP 189 mg/dL, PCT 6.15 ng/mL) with consumption coagulopathy (PT and PTT not detectable, D-Dimer > 69.000), even though the blood culture, pleural fluid culture, and cerebrospinal fluid culture were negative. Therapy based on inotropes and diuretics to treat heart failure, beta blocker and ACE inhibitor to control arterial hypertension, and blood components transfusion to manage coagulopathy was administered. Continuous monitoring with serial echocardiography was performed. A progressive left ventricular hypertrophy due to pressure overload was recognized, with marked diffuse hyperechogenicity of the vascular walls of the aorta, pulmonary, and coronary arteries, without clinical evidence of pulmonary hypertension. He underwent cerebral ultrasounds, which did not show any hemorrhagic or ischemic events. Despite the progressive clinical improvement, his blood pressure gradually increased (90/42 mmHg >> 2DS) with a worsening of the renal functions (creatinine > 2.5 mg/dL). Pharmacological interventions in order to reduce the systemic blood pressure to normal values led to a decrease of the diuresis, which restarted only in the presence of arterial pressure >2DS. A renal ultrasound excluded changes in the renal parenchyma and stenosis of the renal arteries. Thyroid hormones, vanillylmandelic acid, and cortisol were in range, while renin and aldosterone were markedly elevated. The newborn was extubated at 17 days of life, with normalization of the inflammatory parameters and negative cultures. After the extubation, he was breastfed in combination with parenteral nutrition with a good tolerance. Approximately 12 days later, he exhibited sudden clinical worsening with septic shock (CRP 206 mg/dL, PCT 6.1), ascites, decrease of the blood pressure, and anuria. An echocardiography showed a hypokinetic and dilated left ventricle with hyperechoic appearance of large vessels and coronary arteries. The ECG exam was compatible with severe ventricular hypertrophy (strain pattern-type). The combination of severe arterial hypertension, left ventricular hypertrophy, and vascular hyperechogenicity led us to investigate a possible underlying etiology, considering genetic diseases, including GACI, that could explain the entire clinical presentation. Blood samples were collected for molecular investigations and karyotype. The latter resulted normal (46, XY). Despite the beginning of treatment with bisphosphonate, the patient died at 50 days of age due to progressive heart failure with severe systemic hypertension unresponsive to medical treatment that resulted in a fatal acute myocardial infarction. Autopsy was requested.

## 3. Materials and Methods

### 3.1. Autopsy

The autopsy was performed according to national government protocols [7] and common international practices [8]. The law in Italy allows the use and publication of studies on autoptic specimens after removing identifiable details and without a specific approved ethics committee [9]. Furthermore, the autopsy execution does not require parental authorization [10].

### 3.2. DNA Extraction

DNA was extracted from peripheral blood using the QIAamp DNA blood midi Kit (Qiagen, Hilden, Germany; kit code 51185).

### 3.3. Libraray Preparation, Sequencing and Variant Analysis

SureSelect QXT Transposase-Based Library Preparation technology was used for creating the panel library containing the *ENPP1* gene. Enrichment of all coding exons and adjacent intronic regions of the *ENPP1* gene was achieved using the Agilent SureSelect Target Enrichment method for generating an Illumina platform-specific paired-end multiplexed library (according to the Agilent SureSelect/QXT protocols; Agilent Technologies, Santa Clara, CA, USA). Initially, genomic DNA was processed with the Agilent QXT/SureSelect hybrid capture method, and coding regions with adjacent intronic regions of the ENPP1 gene were subsequently enriched by PCR (according to the Agilent SureSelect/QXT protocols). Sequence analysis was then conducted by massive parallel sequencing using the MiSeq Benchtop System (Illumina, San Diego, CA, USA). Bioinformatic analysis was executed using the MiSeq LRM (Illumina) for fastq generation, and a variant analysis was carried out using SeqNext Software version 5 (JSI Medical Systems GmbH, Ettenheim, Germany). Quality criteria require a minimal coverage of 30× for 100% of target regions of the investigated *ENPP1* gene. An average coverage of 1196 sequences per base in the entire target region of the panel was achieved. For assessment and interpretation of the variants, the dbSNP, ExAc, gnomAD, HGMD, LOVD, and UniProt databases, as well as the prediction programs SIFT, PolyPhen2, and Mutationtaster, were used.

## 4. Results

### 4.1. Autopsy Examination

**Figure 1:**
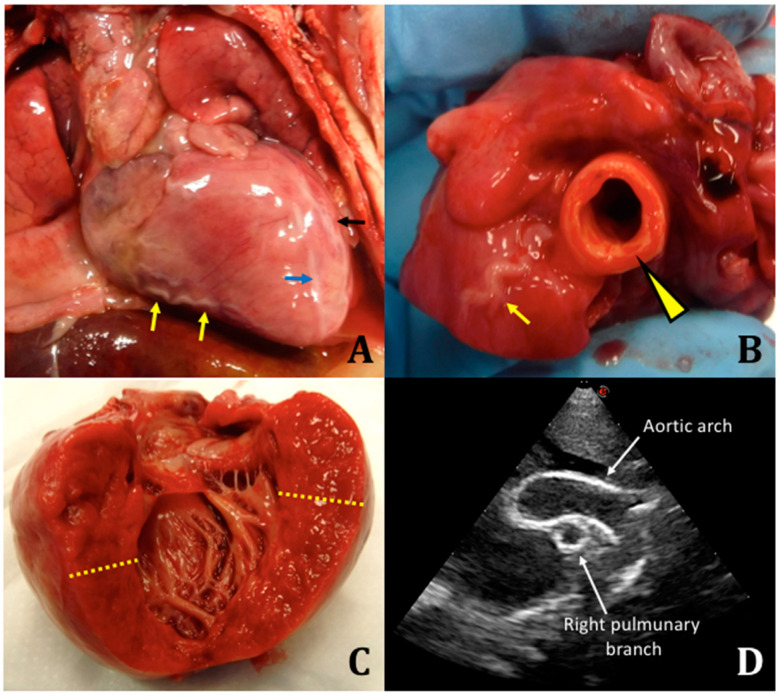
(**A**) Anterior surface of the heart at autopsy. Coronary arteries are thickened, whitish, and sometimes serpiginous. Yellow arrows: right marginal branch of the right coronary artery; blue arrow: left anterior descending artery; black arrow: left marginal artery. (**B**) Detail of the ascending tract of the aorta; the wall appears thickened (arrowhead). On its side, it can be observed the first tract of the right coronary artery thickened, whitish, and serpiginous (arrow). (**C**) Left heart ventricle. The yellow lines highlight the marked hypertrophy of the wall. (**D**) Aortic arch and right pulmonary artery on a postnatal cardiac ultrasound.

We performed an autopsy with fresh and frozen samples collected for molecular testing. Macroscopically, there were no malformations or somatic dysmorphisms. Specifically, we reported peritoneal effusion at the opening of the abdomen (approximately 10 mL), marked cardiomegaly, dilated aorta (1-cm diameter) compared to the pulmonary artery (0.5-cm diameter), enlarged cardiac silhouette, serpentine coronary arteries, left ventricle with thickened wall, atrial septal defect, tricuspid regurgitation, right ventricle with thickness of 4 mm, left ventricle with thickness of 1.1 cm, splenomegaly, flattened adrenal gland, and stiff and calcified aorta at the level of the celiac trunk (Figure 1).

**Figure 2:**
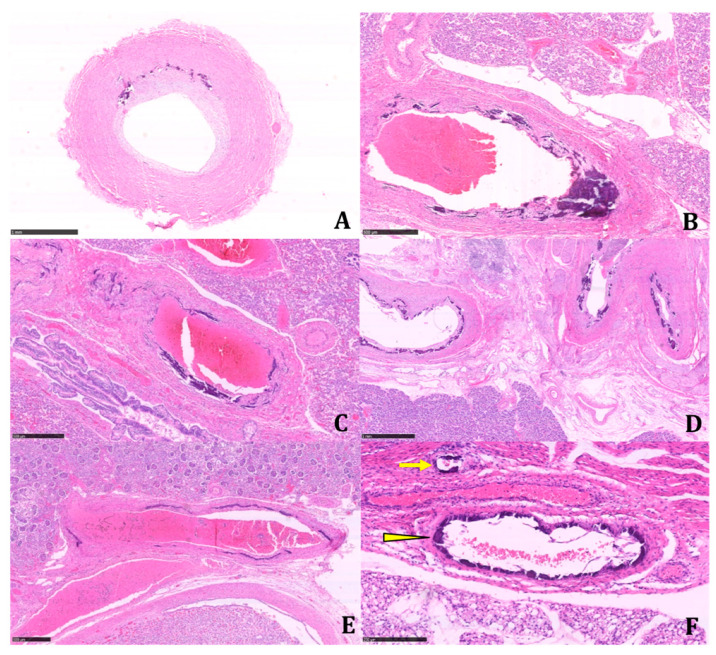
Systemic involvement of large arterial vessels; evidence of calcified vessels next to others histologically not involved. This demonstrates a heterogeneous vascular involvement in the same anatomical site. (**A**) Aorta (H&E, Hematoxylin and eosin; 2.91×). (**B**,**C**) Large hilar and intraparenchymal pulmonary vessels (H&E; 4.84× and 5.15×). (**D**) Retroperitoneal peripancreatic arteries (H&E; 2.22×). (**E**) Renal hilar artery (H&E; 3.79×). (**F**) Retroperitoneal artery of medium caliber (arrowhead) and small caliber (arrow) (H&E; 12.6×). All histological preparations were digitized and photographed with Nanozoomer 360, Hamamatsu. Scale bars: A: 1 mm; B: 500 µm; C: 500 µm; D: 1 mm; E: 500 µm; F: 250 µm).

**Figure 3:**
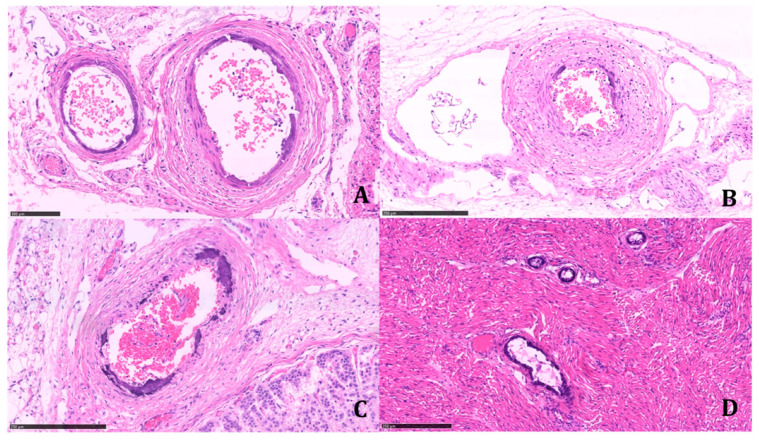
Systemic involvement of small arterial vessels. (**A**) Periaortic retroperitoneal vessels (H&E; 23.1×). (**B**) Retroperitoneal artery interposed between the adrenal gland and kidney (H&E; 16×). (**C**) Periadrenal artery (H&E; 17.9×). (**D**) Intramuscular arteries of various calibers (ileo-psoas muscle) (H&E; 13×). All histological preparations were digitized and photographed with Nanozoomer 360, Hamamatsu. (Scale bars: A: 100 µm; B: 250 µm; C: 250 µm; D: 250 µm).

The histologic examination of the tissue samples revealed arterial dystrophic calcification in the entire length of the aorta, pulmonary arteries, carotids, renal arteries, coronary, periadrenal arteries, bronchial arteries, and in the intramuscular arterioles of the skeletal muscles and peritoneal vessels (Figure 2 and Figure 3).

**Figure 4:**
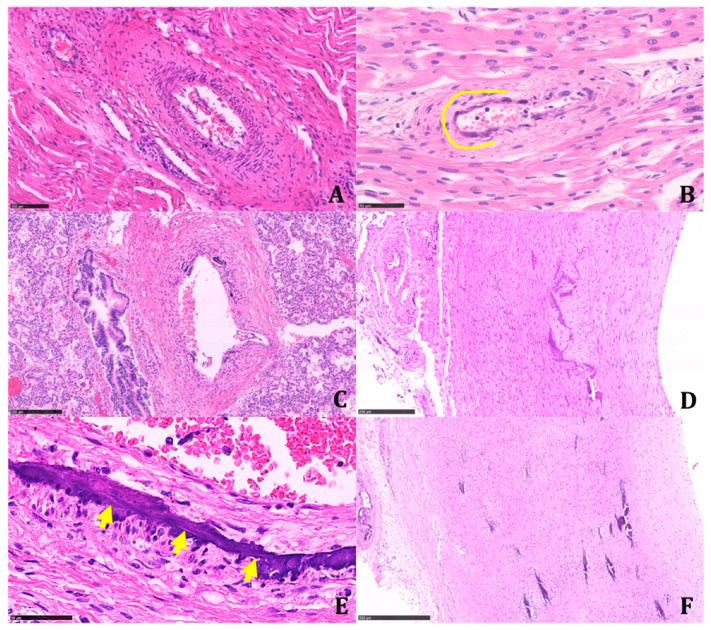
Different degrees of vascular calcified lesions and their intravascular localization observed in the same patient. The simultaneous presence of early and late-stage lesions suggests that this disease changes over time. (**A**) Vessels histologically not affected by calcifications (H&E; 19.2×). (**B**) A small intramyocardial artery with minimal subendothelial calcifications (along yellow line) (H&E; 40×). (**C**) Intrapulmonary artery with initial gross calcifications of the internal elastic lamina (H&E; 10.3×). (**D**) Thoracic aorta with calcifications interposed between the muscular wall and the thick neointima; calcifications extend into the latter and minimally into the muscularis tunica (H&E; 11.2×). (**E**) Renal artery; details of parietal calcification in the inner elastic lamina: the arrows indicate its presence as a thin, purple line (H&E; 61.7×). (**F**) Diffuse calcifications throughout the thickness of the aortic wall (H&E; 7.17×). All histological preparations were digitized and photographed with Nanozoomer 360, Hamamatsu. (Scale bars: A: 100 µm; B: 50 µm; C: 250 µm; D: 250 µm; E: 50 µm; F:500 µm).

Calcifications were coarse, often circumferentially distributed, and mostly identified in the internal elastic membrane, involving both the muscular layer and the neointima (Figure 4).

The latter appeared particularly prominent in large- and medium-sized arteries, with an obliterating effect on the vascular lumen. We found a widespread extension of calcifications, including small systemic arteries, particularly in the skeletal muscle.

**Figure 5:**
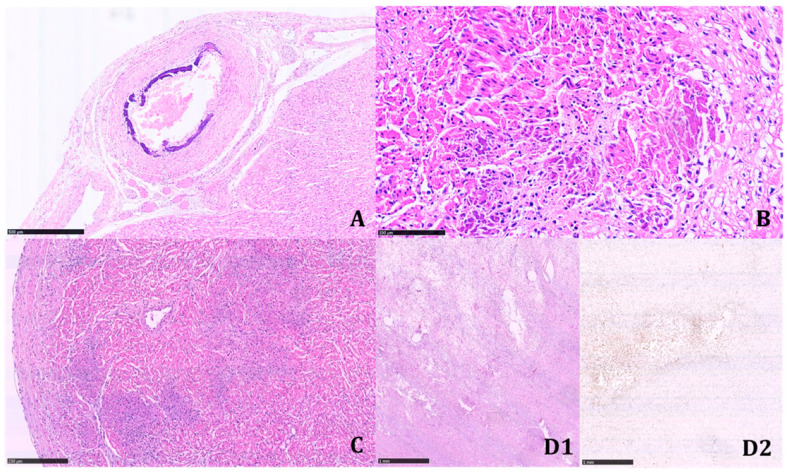
Ischemic visceral changes during GACI. (**A**) Extensively calcific coronary vessel (H&E; 6.29×). (**B**) Cardiac left ventricle: intramyocardial fibrocalcific area; arrows indicate some extravascular calcifications (H&E; 28.5×). (**C**) Left cardiac ventricle; the purple areas indicate myocardial ischemic degeneration (H&E; 10.3×). (**D1**) Brain white matter: infarction in intermediate stage of development (upper left area) (H&E; 2.18×). (**D2**) Same area of the previous image; the immunohistochemical staining for CD68 identifies the marked macrophagic infiltration in the area with ischemic necrosis (CD68-KP1; 2.18×). All histological preparations were digitized and photographed with Nanozoomer 360, Hamamatsu. (Scale bars: A: 500 µm; B: 100 µm; C: 250 µm; D1: 1 mm; D2: 1 mm).

Multinucleated giant cells were not observed where dystrophic calcifications were detected. Marked biventricular myocardial hypertrophy was identified, also involving the septum associated with multiple small foci of myocardial infarction in different stages of development. The foci of infarction were irregularly distributed and localized both in the subepicardial and subendocardial regions. Multiple cerebral infarctions in different stages of development were found (Figure 5).

Bilateral adrenal ectopia in the testes was reported, as well as hyperplasia of the islets of Langerhans of the pancreas. The autopsy findings were highly suggestive of GACI.

### 4.2. Molecular Results

After informed consent was obtained from the parents, blood samples collected from the newborn before death were sent to a specialized laboratory for a molecular analysis of the *ENPP1* gene.

The genetic investigations identified two compound heterozygous variants in the *ENPP1* gene (NM_006208.2): c.1412A>G (p.Tyr471Cys) and c.1715T>C (p. Leu572Ser).

Given the confirmed diagnosis of an autosomal recessive disease, the genetic investigation was extended to the parents to offer adequate counseling regarding future reproductive risks. The c.1412A>G (p.Tyr471Cys) variant was inherited from the mother, and the c.1715T>C (p.Leu572Ser) variant was inherited from the father.

The maternal c.1412A>G (p.Tyr471Cys) variant has been previously reported, and it is classified as pathogenic [11], whereas the paternal variant c.1715T>C (p.Leu572Ser) has currently not been described. The c.1715T>C (p.Leu572Ser) variant is located in the catalytic domain of *ENPP1*, is absent from population databases (like GnomAD), is predicted as damaging by in silico prediction tools [12,13], and is classified as likely pathogenic according to the ACMG guidelines (PM2, PM3, PP3, and PP4) [14].

## 5. Discussion

Generalized Arterial Calcification of Infancy is a rare autosomal recessive disease diagnosed in the presence of cardiovascular symptoms (heart failure, respiratory distress, edema, cyanosis, hypertension, and/or cardiomegaly) in infants with diffuse arterial calcifications and the narrowing of large- and medium-sized vessels not explained by other causes. The identification of biallelic pathogenic variants in the *ENPP1* gene (GACI, type 1) or in the *ABCC6* gene (GACI, type 2) confirms the diagnosis [5].

The patient we report on was clinically suspected and diagnosed of having GACI. The diagnosis was confirmed by autopsy.

The molecular analysis identified two compound heterozygous variants in the *ENPP1* gene (NM_006208.2), one of the two genes related to GACI disease (OMIM#173335; GACI1): c.1412A>G (p.Tyr471Cys) (rs148462924) and c.1715T>C (p.Leu572Ser).

The heterozygous variant c.1412A>G (p.Tyr471Cys) has been described as pathogenic in several unrelated patients with GACI or related diseases [11,15,16,17].

The heterozygous variant c.1715T>C (p.Leu572Ser) has not yet been described in the literature and in the mutation databases. There were no entries for the variation in the 1000Genome project and the Exome Aggregation Consortium, which indicated a very low global minor allele frequency (MAF); it was also not listed in GnomAD or in HGMD. The prediction programs PolyPhen-1 and Mutationtaster classify the variant as damaging. Similar variants have been described in GACI patients (c.1709A>G (p.Y570C)) [1,12]; (c.1737G>C (p.L579F)) [18].

The segregation of the two variants (one inherited from the father and one inherited from the mother), together with the pathognomonic autoptic findings, confirmed the diagnosis of GACI in this patient. The newborn had two siblings from a different father. The parents did not request genetic testing of them. The autopsy findings confirmed that the novel *ENPP1* variant is associated with GACI type 1, at least when found in a compound heterozygous with the c.1412A>G (p.Tyr471Cys) variant. In fact, the autopsy demonstrated a thickening and stiffness of the arterial walls due to intimal proliferation and calcium deposition in the tunica media of the entire aorta, coronary arteries, kidney arteries, pulmonary artery, and its main branches. The histological examination of the myocardium also confirmed the presence of multiple areas of infarction and severe myocardial hypertrophy.

Diffuse and massive calcifications involving not only large- and medium-sized arteries but, also, small-sized arteries were documented. 

There are only a few and nonconsistent data regarding adequate therapy in the literature. In a multicenter genetic study and retrospective observational analysis of 55 individuals affected by GACI, Rutsch F. et al. reported that bisphosphonate therapy is recommended, but it is difficult to understand its effectiveness. Bisphosphonates help in the resolution of calcifications, though they do not affect myointimal proliferation, which plays an important role in narrowing of the vessels [11].

In GACI animal models, the use of magnesium and recombinant ENPP1 enzyme replacement therapy has been reported to have a beneficial effect [3]. 

Prenatal diagnosis and preimplantation genetic diagnosis are possible options for future pregnancies for families with a confirmed molecular diagnosis of GACI in a newborn (25% sibling recurrence risk).

## 6. Conclusions

Generalized Arterial Calcification of Infancy (GACI) is a rare multisystemic disease inherited in an autosomal recessive manner with a wide range of clinical presentations. Given its poor prognosis, an early diagnosis is crucial. 

It is of fundamental importance to improve the knowledge of the genetic mechanisms related to this disease and the correlation between genetic variants and patient phenotype in order to reach a more accurate diagnosis to start early treatment and to provide correct counseling to the parents for future pregnancies. 

The importance of performing genetic analyses is continuously increasing and takes into account the possible impact on clinical management and natural progression of the disease. 

Further studies are needed to expand the genetic spectrum of this rare pathology.

## Data Availability

Management System of the newborn at Santa Chiara Hospital, Trento. Data directly not available due to privacy.
